# Thomas J Henry: longtime friend, colleague, and preeminent heteropterist

**DOI:** 10.3897/zookeys.796.30926

**Published:** 2018-11-15

**Authors:** Alfred G. Wheeler, Jr.

**Affiliations:** 1 Department of Plant and Environmental Sciences, Clemson University, Clemson, SC 29634-0310, USA Clemson University Clemson United States of America

Tom Henry came to the Pennsylvania Department of Agriculture, Bureau of Plant Industry (Harrisburg), in 1972. He recently had earned a B.S. degree in Entomology from Purdue University and was hired to identify insects, mainly those submitted by the bureau’s plant inspectors in different areas of the state. It did not take long for his entomological colleagues in Harrisburg to recognize his remarkable talents as a taxonomist.

I had been hired the previous year to work on bionomics of insects associated with ornamental trees and shrubs and had collected a mirid on Scots pine (*Pinussylvestris* L.), which had defied identification. Tom accepted the challenge of trying to identify the mystery plant bug. A trip to examine Miridae in the National Museum of Natural History in Washington, DC, resolved the issue: the conifer bug was *Camptozygumaequale* (Villers), a species new to North America (Wheeler and Henry 1973). To become more familiar with the local mirid fauna, we began to spend our lunch breaks sampling conifers and other trees in nearby ornamental plantings. The detection of *C.aequale* was followed by the discovery of additional Palearctic conifer-feeding plant bugs in Pennsylvania. Tom soon described his first new species: two pine-associated mirids of the diverse genus *Phytocoris* (Henry 1974). Gaining confidence and momentum, he published 27 additional papers on Heteroptera by decade’s end, including a review of the orthotyline genus *Reuteria* (Henry 1976), ten new species of the Neotropical mird genus *Hyalochloria* (Henry 1978), a new genus and species of cardiastethine Anthocoridae (Henry and Herring 1978), and new species of the isometopine genus *Corticoris* (Henry and Herring 1979).

Tom’s entry into heteropteran systematics caught the attention of prominent North American specialists, such as Richard Froeschner, John Lattin, and James Slater. He was encouraged to apply for the Heteroptera position with the Systematic Entomology Laboratory (SEL), Agricultural Research Service, US Department of Agriculture, which had become available with J. L. Herring’s retirement in August 1979. In selecting Tom for the position, the search committee must have valued his publication record and recognized his taxonomic prowess; committee members were not dissuaded by his lack of the ordinarily requisite PhD (he was finishing his MS studies at Penn State). The decision to hire Tom Henry proved auspicious for SEL.

Tom began his work at SEL in June of 1980. Productivity characterized his performance in the 1980s. He continued taxonomic research on plant bugs of the Orthotylinae and on the subfamily Isometopinae, describing three new genera (Henry 1980). Highlighting the decade was the 1988 publication of the first catalog of North American Heteroptera since 1917. Henry and Froeschner (1988) accomplished what other heteropterists had attempted to do but ultimately found too daunting. A novel feature of their catalog, which some systematists initially considered inappropriate, was the use of write-ups and photos of representative species at the beginning of each family treatment. Biological control specialists, ecologists, and others not well versed in heteropteran taxonomy found the introductory material particularly valuable. His early trips for fieldwork and visits to major insect collections eventually involved nearly all U.S. states, Asia, Australia, Canada, Europe, India, Mexico, South America, and the West Indies.

Tom continued to prosper in the 1990s despite pursuing a doctoral degree while working full-time. He received his PhD in 1995 from the University of Maryland. Prominent among his accomplishments during the decade were taxonomic revisions of mirid genera (Henry 1991, 1994) and a phylogenetic analysis of the infraorder Pentatomomorpha, involving 34 family groups, which resulted in the recognition of 11 families in a previously composite and paraphyletic Lygaeidae (Henry 1997a). His substantial reclassification of the Pentatomomorpha now is followed in text books, world catalogs, and applied and taxonomic literature. He emphasized the family Berytidae, completing three major works: a cladistic analysis and revision of world genera (Henry 1997b), a monograph of the stilt bugs of the Western Hemisphere (Henry 1997c), and a catalog of the world species (Henry and Froeschner 1998). Tom coauthored a book on the North American mirids considered naturally Holarctic or adventive, either immigrant or intentionally introduced (Wheeler and Henry 1992), and edited a Festschrift of approximately 250 pages that honored the renowned Brazilian miridologist José Carvalho (Henry and Wheeler 1995).

Perusal of the appended list of Tom’s publications will confirm his prominence among world heteropterists. Collaborative projects with other specialists and promising newcomers became routine in the new century (e.g., Henry and Schuh 2002, Henry and Costa 2003), along with more generic revisions (Henry 2006; Ferreira and Henry 2010; Henry 2012, 2015, 2018; Dellapé et al. 2016). Key contributions included the berytid chapter (Henry 2000) in *Heteroptera of Economic Importance* (Schaefer and Panizzi 2000), a comprehensive treatment of Cuban Miridae (Hernández and Henry 2010), chapters on the Miridae (Ferreira et al. 2015) and Lygaeoidea (Henry et al. 2015) in *True Bugs (Heteroptera) of the Neotropics* (Panizzi and Grazia 2015), and an online world catalog of the Lygaeoidea (Dellapé and Henry 2017).

A typical work day for Tom includes a delivery of “urgents”, specimens of Heteroptera intercepted at US ports of entry that need to be identified promptly. He also curates the collection under his care and supervises technicians and volunteers who work with the collection. Tasks expected of a professional entomologist are dealt with promptly, such as reviewing manuscripts at the request of journal editors, providing pre-submission manuscript Book reviews for colleagues and writing letters in support of their tenure, serving on committees, and participating in the affairs of scientific societies. Tom edited the *Proceedings of the Entomological Society of Washington* from 1992 to 1995 and began a second stint as editor in 2015, which continues. He is a founding member of the International Heteropterists’ Society and will become President at its 6^th^ quadrennial meeting in La Plata, Argentina, in December 2018. He is generous with his time in encouraging and mentoring younger workers and assisting students and established specialists who visit the Heteroptera collection. Tom and his wife Kathryn (Katy) often invite visitors to their home for a meal.

How is Tom able to be so productive? For one thing, he possesses a sterling work ethic. He’s efficient, passionate about natural history and systematics, and strives for excellence. A typical day begins at 4:30 am; he works at home for an hour or two on his own manuscripts or Book reviews those of others, before he leaves for the museum. He is focused, and patient, even though he often must stop what he is doing to answer visitors’ questions or respond to e-mails and phone requests.

Numerous projects on Heteroptera await Tom’s attention. We can anticipate a flurry of papers in the coming years. It is a pleasure to present this special issue of *ZooKeys* that conveys the respect of his colleagues and honors his 70^th^ birthday.
